# Impact of Robotic Assistance and Fixation Type on Revisions, Complications, and Survivorship Following Total Knee Arthroplasty

**DOI:** 10.2106/JBJS.OA.26.00043

**Published:** 2026-05-06

**Authors:** Hania Shahzad, Mandeep S. Chhokar, Sahej Deep Singh Randhawa, John P. Meehan, Mauro Giordani, Adam J. Taylor, Zachary C. Lum

**Affiliations:** 1 Department of Orthopaedic Surgery, UC Davis Health, Sacramento, California, USA

## Abstract

**Introduction:**

With the increasing adoption of both cementless fixation and robotic-assisted techniques in total knee arthroplasty (TKA), it is important to recognize that these approaches carry distinct historical concerns. Earlier generations of cementless implants were associated with higher early failure rates due to inadequate osseointegration, while early robotic systems faced criticism for increased operative times and complication risk. As a result, the current literature presents conflicting evidence regarding the impact of these technologies on early complications and revision rates. This study compares the reoperation rates across 4 TKA cohorts, cemented vs. cementless, with and without robotic assistance.

**Methods:**

A retrospective cohort study was conducted using a national administrative claims database. Primary TKA cases were identified using International Classification of Diseases, 10th Revision, and Current Procedural Terminology codes, and 4 cohorts were created: robotic-cemented (R-CEMENT), robotic-cementless (R-CEMENTLESS), conventional-cemented (C-CEMENT), and conventional-cementless (C-CEMENTLESS). Matching was performed based on age, sex, Elixhauser Comorbidity Index, obesity, tobacco use, and diabetes, resulting in 5,210 patients in each group. Outcomes assessed included 1-, 5-, and 10-year ipsilateral reoperations, 30-day emergency department utilizations, and 10-year failure-free survival. χ^2^ tests were used for group comparisons, with p < 0.05 indicating significance.

**Results:**

In the matched cohort (n = 20,840, C-CEMENTLESS, C-CEMENT, R-CEMENTLESS, R-CEMENT; n = 5,210 each), 1-year reoperation rates were lowest in C-CEMENTLESS (0.44%) and R-CEMENTLESS (0.52%), followed by R-CEMENT (0.84%) and C-CEMENT (0.92%) (p = 0.005). At 5 and 10 years, reoperation rates remained lowest in C-CEMENTLESS (0.79%) and R-CEMENTLESS (1.02%), compared with higher rates in C-CEMENT (1.86%) and R-CEMENT (1.71%) (p < 0.001). Kaplan–Meier survival showed 99.7% 5-year survivorship in C-CEMENTLESS and R-CEMENTLESS, versus 99.5% in both cemented groups (p-value < 0.05). No significant differences were observed in 30-day ED utilization (p = 0.11) or readmissions (p = 0.75) across all 4 matched cohorts.

**Conclusion:**

Cementless fixation in TKA, whether robotic or conventional, demonstrated comparable short-term reoperation rates and equivalent long-term survivorship to cemented fixation. Robotic assistance did not significantly affect failure-free survival or healthcare utilization. All 4 cohorts showed excellent 10-year outcomes.

## Introduction

The long-term implant survivorship of total knee arthroplasty (TKA) exceeds 95% at 10 years postoperatively^1-3^. Historically, cementless fixation in total knee arthroplasty raised concerns due to higher early failure rates seen in the 1990s and early 2000s, largely attributed to insufficient osseointegration of the tibial and femoral components. When osseointegration did occur, long-term survivorship was generally excellent; however, early failures related to lack of biologic fixation limited widespread adoption at that time^4,5^. Consequently, cemented fixation remains the gold standard in primary TKA; however, cementless fixation has seen renewed interest due to advancements in implant design and promising short-term outcomes, including lower revision rates in selected patient populations^6-8^. At the same time, technology-assisted arthroplasty, particularly robotic-assisted TKA (Ra-TKA), has rapidly gained popularity, with over 70% of surveyed American Association of Hip and Knee Surgeons members reporting its use to enhance surgical precision, alignment, ligament balance, and implant positioning^9^. There remains a paucity of data examining outcomes when these techniques are combined, and some evidence is conflicting on whether the use of technology or cementless implants does indeed have benefits. In addition, existing literature often treats robotic and nonrobotic techniques as homogeneous groups, without stratifying outcomes based on fixation method. This represents a critical knowledge gap, as robotic platforms may impact the success of cementless fixation through more accurate bone preparation and soft tissue balancing^10-12^.

The objective of this study was to compare the long-term reoperation rates, failure-free survival, and emergency department utilization among patients undergoing primary TKA across 4 cohorts: cemented vs. cementless fixation in both conventional and robotic-assisted techniques. By analyzing these groups, this study aims to explore whether robotic assistance modifies the risk profiles traditionally associated with each fixation method, thereby guiding surgical decision-making in the era of personalized arthroplasty.

## Methods

A retrospective cohort study was conducted using a national administrative claims database (PearlDiver Technologies, Colorado Springs) containing a total of 170 million records between 2015 and 2024. Primary TKA cases (Current Procedural Terminology [CPT]-27447) were identified using International Classification of Diseases, 10th Revision (ICD-10) and CPT codes to classify procedures as robotic (CPT-S29001; ICD-10-P: 8E0Y0CZ, 8E0Y3CZ, 8E0Y4CZ, 8E0YXCZ), cemented (ICD-10-P: 0SRC069, 0SRC0J9, 0SRC0L9, 0SRC0M9, 0SRC0N9, 0SRD069, 0SRD0J9, 0SRD0L9, 0SRD0M9, 0SRD0N9), and not-cemented (ICD-10-P-0SRC06A, ICD-10-P-0SRC0JA, ICD-10-P-0SRC0LA, ICD-10-P-0SRC0MA, ICD-10-P-0SRC0NA, ICD-10-P-0SRD06A, ICD-10-P-0SRD0JA, ICD-10-P-0SRD0LA, ICD-10-P-0SRD0MA, ICD-10-P-0SRD0NA).

### Matching

Four cohorts were created: robotic-cemented (R-CEMENT), robotic-cementless (R-CEMENTLESS), conventional-cemented (C-CEMENT), and conventional-cementless (C-CEMENTLESS), ensuring that there is no overlap of patients across each cohort. Matching was performed based on age, sex, Elixhauser Comorbidity Index (ECI), obesity, tobacco use, and diabetes. The ECI is one of the indices used to quantify comorbidity burden in both surgical and database research. It has been shown to outperform the Charlson Comorbidity Index (CCI) in predicting 8 of the 18 complications analyzed and was not inferior in predicting any complication^13^. After matching, each cohort comprised 5,210 patients.

### Outcomes

Primary outcomes assessed included 1-, 5-, and 10-year ipsilateral reoperations, 30-day emergency department visits, and 30-day ED-related hospital readmissions. Reoperation rates were defined as any incidence of revision on the ipsilateral side, identified using CPT codes 27486 and 27487, or the following ICD-9 and ICD-10 procedure codes: ICD-9-P-0080, 0081, 0082, 0083, 0084, 8155; and ICD-10-P-0SWC04Z, 0SWC08Z, 0SWC09Z, 0SWC0JC, 0SWC0JZ, 0SWC4JZ, 0SWCX5Z, 0SWCX8Z, 0SWCXJC, 0SWCXJZ, 0SWD04Z, 0SWD08Z, 0SWD09Z, 0SWD0JC, 0SWD0JZ, 0SWD4JZ, 0SWDX8Z, 0SWDXJZ, 0SWT0JZ, 0SWTXJZ, 0SWU0JZ, 0SWUXJZ, 0SWV0JZ, 0SWVXJZ, 0SWW0JZ, and 0SWWXJZ that corresponds to revision procedures. For the causes of reoperations, a new bucket was created containing all diagnosis records linked to the claim IDs in the primary bucket. The included diagnosis records were filtered based on diagnosis position and/or admitting diagnoses, allowing the construction of a dataset of codes representing conditions present at the time of the event (e.g., repeat TKA on the ipsilateral side). All associated diagnoses were categorized into 6 themes based on corresponding ICD-9/10 codes: infection, degenerative conditions, pain and other symptoms, traumatic conditions, mechanical complications, and implant-related complications (Table I).

**TABLE I:** Thematic Classification of Diagnoses Associated with Reoperations Based on ICD-9/10 Codes

A. Infection	
Code ICD-10-D-L03115	Cellulitis of right lower limb
Code ICD-10-D-M00062	Staphylococcal arthritis left knee
Code ICD-10-D-M00861	Arthritis due to other bacteria, right knee
Code ICD-10-D-M00862	Arthritis due to other bacteria, left knee
Code ICD-10-D-T8453XA	Infection and inflammatory reaction due to internal right knee prosthesis, initial encounter
Code ICD-10-D-T8453XD	Infection and inflammatory reaction due to internal right knee prosthesis subsequent encounter
Code ICD-10-D-T8454XA	Infection and inflammatory reaction due to internal left knee prosthesis, initial encounter
Code ICD-10-D-T8454XD	Infection and inflammatory reaction due to internal left knee prosthesis subsequent encounter
Code ICD-10-D-T8454XS	Infection and inflammatory reaction due to internal left knee prosthesis sequela
Code ICD-10-D-T8459XS	Infection and inflammatory reaction due to other internal joint prosthesis sequela
B. Degenerative conditions	
Code ICD-10-D-M1711	Unilateral primary osteoarthritis right knee
Code ICD-10-D-M1712	Unilateral primary osteoarthritis left knee
Code ICD-10-D-M1731	Unilateral post-traumatic osteoarthritis right knee
Code ICD-10-D-M1732	Unilateral post-traumatic osteoarthritis left knee
Code ICD-10-D-T84062A	Wear of articular bearing surface of internal prosthetic right knee joint, initial encounter
Code ICD-10-D-T84063A	Wear of articular bearing surface of internal prosthetic left knee joint, initial encounter
Code ICD-10-D-T84063D	Wear of articular bearing surface of internal prosthetic left knee joint, subsequent encounter
Code ICD-10-D-M13861	Other specified arthritis right knee
C. Pain and other symptoms	
Code ICD-10-D-M25552	Pain in left hip
Code ICD-10-D-M25561	Pain in right knee
Code ICD-10-D-M25562	Pain in left knee
Code ICD-10-D-M79662	Pain in left lower leg
Code ICD-10-D-M25661	Stiffness of right knee not elsewhere classified
Code ICD-10-D-M25662	Stiffness of left knee not elsewhere classified
D. Traumatic conditions	
Code ICD-10-D-M12561	Traumatic arthropathy right knee
Code ICD-10-D-M9711XA	Periprosthetic fracture around internal prosthetic right knee joint initial encounter
Code ICD-10-D-M9712XA	Periprosthetic fracture around internal prosthetic left knee joint initial encounter
Code ICD-10-D-S72465A	Nondisplaced supracondylar fracture with intracondylar extension of lower end of left femur, initial encounter for closed fracture
Code ICD-10-D-S76111A	Strain of right quadriceps muscle fascia and tendon initial encounter
Code ICD-10-D-S76112A	Strain of left quadriceps muscle fascia and tendon initial encounter
Code ICD-10-D-S8002XA	Contusion of left knee initial encounter
Code ICD-10-D-S81002A	Unspecified open wound left knee initial encounter
Code ICD-10-D-S83102A	Unspecified subluxation of left knee initial encounter
Code ICD-10-D-S83194D	Other dislocation of right knee, subsequent encounter
Code ICD-10-D-S86812A	Strain of other muscle(s) and tendon(s) at lower leg level, left leg, initial encounter
E. Mechanical complications	
Code ICD-10-D-T84022A	Instability of internal right knee prosthesis initial encounter
Code ICD-10-D-T84023A	Instability of internal left knee prosthesis initial encounter
Code ICD-10-D-T84023D	Instability of internal left knee prosthesis subsequent encounter
Code ICD-10-D-T84032A	Mechanical loosening of internal right knee prosthetic joint initial encounter
Code ICD-10-D-T84032D	Mechanical loosening of internal right knee prosthetic joint, subsequent encounter
Code ICD-10-D-T84033A	Mechanical loosening of internal left knee prosthetic joint initial encounter
Code ICD-10-D-T84092A	Other mechanical complication of internal right knee prosthesis initial encounter
Code ICD-10-D-T84093A	Other mechanical complication of internal left knee prosthesis initial encounter
Code ICD-10-D-T84093D	Other mechanical complication of internal left knee prosthesis, subsequent encounter
Code ICD-10-D-M2351	Chronic instability of knee right knee
Code ICD-10-D-M2352	Chronic instability of knee left knee
Code ICD-10-D-M238X1	Other internal derangements of right knee
Code ICD-10-D-M24562	Contracture left knee
Code ICD-10-D-M24661	Ankylosis right knee
Code ICD-10-D-M24662	Ankylosis left knee
Code ICD-10-D-M25061	Hemarthrosis right knee
Code ICD-10-D-M25361	Other instability right knee
Code ICD-10-D-M25362	Other instability left knee
Code ICD-10-D-M25461	Effusion right knee
F. Implant or device-related complications	
Code ICD-10-D-T84012A	Broken internal right knee prosthesis initial encounter
Code ICD-10-D-T84012D	Broken internal right knee prosthesis subsequent encounter
Code ICD-10-D-T84013A	Broken internal left knee prosthesis initial encounter
Code ICD-10-D-Z89521	Acquired absence of right knee
Code ICD-10-D-Z96651	Presence of right artificial knee joint
Code ICD-10-D-Z96652	Presence of left artificial knee joint

ICD-10 = International Classification of Diseases, 10th Revision.

### Provider Metrics

We conducted a retrospective analysis using the PearlDiver database (PearlDiver Technologies) to evaluate provider-level metrics for procedural volume and associated outcomes. Owing to the discontinuation of provider-specific data in the most recent release (updated through 2024, containing 175 million patient records), we used an earlier version of the database comprising 160 million patient records, which retained provider-level reporting. The provider report tool generated spreadsheets detailing the number of patients and records per provider, average and median procedures per provider, length of stay, charges, reimbursements, and facility-specific geographic information (city, state, ZIP code, and metropolitan statistical area) per day. Procedural cohorts were defined using standardized CPT and ICD-10 code groupings, and descriptive statistics were calculated to quantify average and median procedures per provider across buckets. This analysis provided insights into provider experience and volume, supporting the interpretation of outcomes discussed in the results.

### Kaplan-Meier Survival Analysis

A retrospective survival analysis was conducted to evaluate time to reoperation following TKA. Kaplan-Meier curves were generated for patients identified in the “TKA” cohort. The “start event” was defined as the “first instance of TKA” on a given side. The “end event” was defined as the “first subsequent reprocedure on the same side”. Patient survival time was calculated from the date of the index TKA to the earliest occurrence of: (1) a repeat TKA procedure on the ipsilateral as defined by the codes mentioned previously, (2) 10 years of follow-up, or (3) loss to follow-up or dataset inactivity, whichever occurred first. Survival probabilities were plotted to compare the durability of revision-free implants over time.

### Statistical Analysis

χ^2^ tests were used for group comparisons, with p < 0.05 indicating significance.

## Results

A total of 20,840 matched patient records were analyzed, with 5,210 included in each of the 4 cohorts for comparative analysis.

### Reoperation Rates

In the matched cohorts (n = 20,840), 1-year reoperation rates for the matched cohort were 0.44% (23) in C-CEMENTLESS, 0.92% (48) in C-CEMENT, 0.52% (27) in R-CEMENTLESS, and 0.84% (44) in R-CEMENT (p = 0.005). 5-year reoperation rates for the matched cohort were 0.79% (41) in C-CEMENTLESS, 1.86% (97) in C-CEMENT, 1.02% (53) in R-CEMENTLESS, and 1.71% (89) in R-CEMENT (p < 0.001). Ten-year reoperation rates in the matched cohort were 0.79% (41) for C-CEMENTLESS, 1.86% (97) for C-CEMENT, 1.02% (53) for R-CEMENTLESS, and 1.71% (89) for R-CEMENT (p <0.001) (Table II)

**TABLE II:** Comparison of 1- and 5-Year Revisions and Short-Term Complications by Fixation Method and Robotic Assistance in Primary TKA

Metric	Conventional		Robotic		p
	Cementless n (%)	Cemented n (%)	Cementless n (%)	Cemented n (%)	
Matched (n)	5,210	5,210	5,210	5,210	
1-year revisions	23 (0.44)	48 (0.92)	27 (0.52)	44 (0.84)	0.005
5-year revisions	41 (0.79)	97 (1.86)	53 (1.02)	89 (1.71)	4.88 × 10^-07^
10-year revisions	45 (0.86)	113 (2.17)	56 (1.07)	91 (1.75)	1.48 × 10^-08^
ED visits	340 (6.53)	397 (7.62)	367 (7.04)	394 (7.56)	0.11
Readmissions	15 (0.29)	16 (0.31)	18 (0.35)	21 (0.40)	0.75
Provider metrics*					
Average procedures per provider	3.6	3.5	5.6	2.5	—
Median procedures per provider	3.0	3.0	5.0	2.0	—

TKA = total knee arthroplasty. *Provider-level metrics derived from a retrospective PearlDiver database analysis (160M records; PearlDiver Technologies, CO) using provider report tools; cohorts defined by CPT and ICD-10 codes and summarized with descriptive statistics.

In the matched cohorts (n = 20,840), the primary diagnosis on all records for ipsilateral reoperation during 10-year follow-up was degenerative conditions (see Table III). When examining admitting diagnoses for reoperations or failures, infection, and mechanical complications were the predominant causes. Infection accounted for 44.4% (20/45) of C-CEMENTLESS, 39.8% (45/113) of C-CEMENT, 35.7% (20/56) of R-CEMENTLESS, and 33.0% (30/91) of R-CEMENT reoperations. Mechanical complications were observed in 37.8% (17/45) of C-CEMENTLESS, 51.3% (58/113) of C-CEMENT, 44.6% (25/56) of R-CEMENTLESS, and 45.0% (41/91) of R-CEMENT reoperations. Other causes, such as traumatic conditions, pain, and implant/device-related complications, were less frequent across all groups (Table III).

**TABLE III:** Causes of Ipsilateral Reoperations During 10-Year Follow-up Across all 4 Cohorts

	Conventional		Robotic	
	Cementless n = 45 (0.86%)	Cemented n = 113 (2.17%)	Cementless n = 56 (1.07%)	Cemented n = 91 (1.75%)
Primary diagnosis*				
Degenerative conditions	43	110	54	91
Admitting diagnosis				
Infection	20	45	20	30
Pain and other symptoms	1	3	1	5
Traumatic conditions	1	4	6	14
Mechanical complications	17	58	25	41
Implant & device-related complications	3	6	5	8

*The sum of causes exceeds the total number of reoperations in each group because multiple diagnoses can be assigned to a single reoperation. For example, a patient undergoing revision for infection may also have mechanical or implant-related complications documented. Therefore, counts by cause are not mutually exclusive and represent diagnosis frequencies rather than distinct cases.

### Kaplan-Meier Survival Analysis

**Fig. 1:**
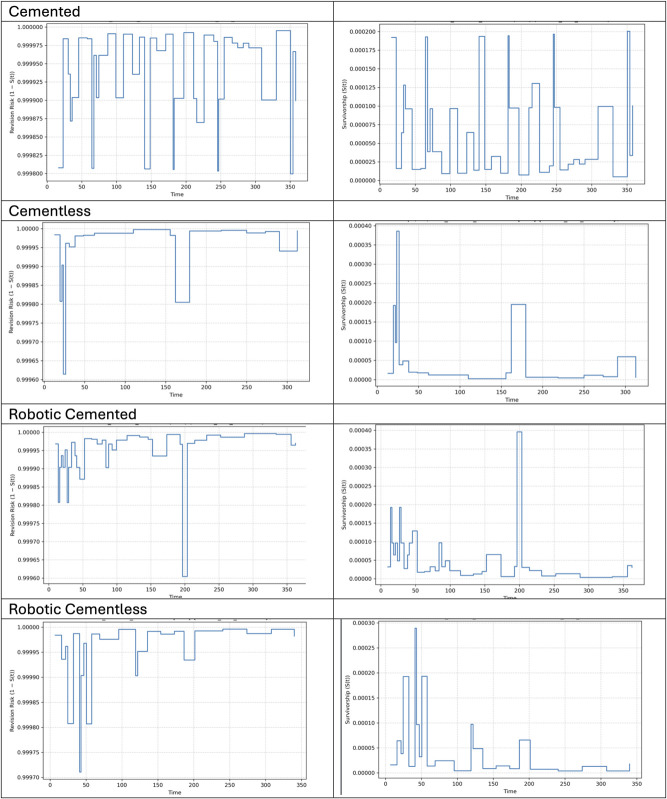
Kaplan-Meier survival curves comparing cemented and cementless total hip arthroplasty using conventional and robotic-assisted techniques.

The Kaplan-Meier survival curves for matched cohorts showed high survivorship across all groups at 5 years, with C-CEMENTLESS and R-CEMENTLESS both demonstrating 99.7% survival, and C-CEMENT and R-CEMENT each showing 99.5%. By 10 years, a small divergence was observed: C-CEMENTLESS and R-CEMENTLESS maintained 99.5% survival, whereas C-CEMENT and R-CEMENT demonstrated slightly lower survivorship at 99.1%. The standard error at 10 years remained low across all cohorts, ranging from 0.0009 to 0.0013, indicating high precision in survival estimates. The 95% confidence intervals for 10-year survival estimates further supported these findings, with C-CEMENTLESS (0.994-0.997) and R-CEMENTLESS (0.993-0.997), demonstrating slightly narrower and higher survival bounds compared with C-CEMENT (0.988-0.993) and R-CEMENT (0.989-0.994) (Table IV and Fig. 1).

**TABLE IV:** Kaplan-Meier Curves Demonstrating Time to Ipsilateral Reoperation for the 4 Matched Study Groups

Group	5-Yr Survival (%)	10-Yr Survival (%)	Standard Error (10 yrs)	95% Confidence Interval	Log-Rank p
C-CEMENTLESS	99.7	99.5	0.000941	0.994-0.997	<0.05
C-CEMENT	99.5	99.1	0.001325	0.988-0.993	<0.05
R-CEMENTLESS	99.7	99.5	0.001013	0.993- 0.997	<0.05
R-CEMENT	99.5	99.1	0.001291	0.989- 0.994	<0.05

### 30-Day Health Care Utilization

In matched cohorts, the incidence of ED visits was 6.53% (340) in C-CEMENTLESS, 7.62% (397) in C-CEMENT, 7.04% (367) in R-CEMENTLESS, and 7.56% (394) in R-CEMENT (p = 0.11). In the matched population, readmissions within 30 days occurred in 0.29% (15) of C-CEMENTLESS, 0.31% (16) of C-CEMENT, 0.35% (18) of R-CEMENTLESS, and 0.40% (21) of R-CEMENT (p = 0.75) patients.

### Provider Metrics

In the C-CEMENTLESS and C-CEMENT cohorts, providers performed a mean of 3.6 (SD 1.5) and 3.5 (SD 2.0) primary TKAs, respectively, with median values of 3.0 in both groups. By contrast, providers in the R-CEMENTLESS cohort performed a higher mean number of procedures, 5.6 (SD 4.0), while those in the R-CEMENT cohort performed fewer procedures on average, 2.5 (SD 1.6), with corresponding median values of 5.0 and 2.0, respectively

## Discussion

This study leveraged a national claims database to evaluate failure rates across different fixation techniques and robotic-assisted navigation across 20,840 matched patients (5,210 per cohort). This study revealed that cementless fixation was associated with a consistently lower incidence of reoperations compared to cemented fixation across both robotic and nonrobotic groups at the 1-, 5-, and 10-year follow-up. The higher proportion of mechanical complications in cemented groups, particularly nonrobotic procedures, may be attributable to fixation technique and the long-term material behavior of cement, which can predispose to late loosening, cement degradation, or periprosthetic fractures. Conversely, the slightly higher rate of traumatic causes in the robotic-assisted cementless group may reflect differences in patient activity levels, surgical selection, or residual confounding despite matching.

Kaplan Meier survival analysis demonstrated clinically equivalent long-term survivorship between fixation types, aligning with recent evidence registry data and supporting cementless fixation as a viable, and potentially superior alternative, particularly in select patient populations^14^.

Robotic-assisted TKA has seen steady growth, with utilization increasing from 0.35% in 2010 to 3.45% in 2022^12^. While it has been shown to improve the accuracy of implant positioning and reduce radiographic outliers, our study found no significant difference in 1- or 5-year failure-free survivorship when comparing robotic with conventional TKA. This finding is consistent with previous studies, including those by Molho et al., which also demonstrated equivalent short-term survivorship between robotic and manual techniques^10^. Notably, most previous studies are limited by single-institution or single-surgeon designs, whereas our study incorporated a nationally representative sample with matched cohorts and adjustment for patient-specific variables. Although we did not assess specific causes for reoperation, our results suggest that robotic assistance may not universally translate to improved early clinical outcomes. Given the significant capital costs, specialized training, and longer operative times associated with robotic platforms^15^, routine use may not be justified in all cases. Instead, robotic-assisted TKA may be best reserved for individualized cases or lower-volume surgeons where enhanced precision in component placement could confer greater benefit.

Our study benefits from matching across multiple variables and adjustment for patient-specific variables, large cohort sizes, and comprehensive comparison across all combinations of fixation techniques and robotic assistance. Nevertheless, limitations include its retrospective design, potential coding inaccuracies inherent to claims databases, and generalizability limited to US populations. The findings of this study do not favor any 1 particular group for survivorship; therefore, future prospective studies are needed to validate these observations and assess their clinical significance.

In addition, the PearlDiver database lacks granular clinical information, including radiographic findings, implant positioning, functional outcomes, and patient-reported outcome measures. As a result, our definition of “reoperation”, derived from procedural codes, may not fully capture modes of early failure such as aseptic loosening, instability, or mechanical complications that are more accurately identified in registry-based studies. Prior national joint registries have reported subtle differences in early failure patterns between cemented and cementless implants, which may not be detectable using claims-based data^11,15^. Therefore, conclusions regarding clinical equivalence should be interpreted with caution.

Furthermore, although matching on comorbidities improves cohort comparability, our analysis is also limited by the absence of data regarding bone quality, and implant design characteristics as well as provider-specific factors such as surgeon experience and potential learning curves. While we have included provider volume metrics, they only offer a glimpse into operative expertise and may not fully encapsulate variation at the surgeon level. Previous work by Kayani et al. has demonstrated that early postoperative outcomes are influenced by surgeon experience rather than fixation technique alone, with a significant learning curve associated with robotic-assisted TKA^16^. Therefore, discrepancies in surgeon proficiency/familiarity with robotic systems may contribute to variation in revision rates and should be considered in conjunction with our findings.

Finally, although our findings indicate that robotic assistance did not confer a survivorship advantage, this result should be interpreted within the limitations of claims-based data. Our results are consistent with recent meta-analytic evidence demonstrating no clear improvement in revision rates with robotic-assisted TKA^15^. Survivorship represents only 1 dimension of performance, and several potential benefits of robotic systems, including reductions in radiographic outliers, improvements in limb and component alignment, greater precision of bone preparation, and short-term improvements in patient satisfaction or functional recovery, are not captured within administrative datasets. Furthermore, there is a lack of information regarding the specific knee replacement implant systems and robotic platforms used, as the PearlDiver database does not report device or system level details. Consequently, we were unable to differentiate outcomes by implant design or robotic system type. Only procedures with CPT and ICD codes corresponding to robotic-assisted TKA were included, but the exact robotic platforms used could not be identified. Therefore, the absence of a revision benefit in this analysis does not preclude meaningful advantages in early functional or radiographic outcomes that may still justify the use of robotic assistance in selected patients.

## Conclusion

The findings of this large national claims database analysis indicate clinically equivalent failure-free survivorship between cemented and cementless techniques over a 10-year period. No clinically meaningful differences were observed in 30-day emergency department utilization among matched cohorts. In addition, the use of robotic assistance did not confer a survival advantage at 1-, 5-, and 10-year intervals. Overall, all cohorts exhibited high survivorship, underscoring the procedural durability of primary TKA regardless of fixation method or robotic utilization.
